# Supplementary figures and images for: Wnt/β-catenin signalling underpins juvenile Fasciola hepatica growth and development
Source: PLoS Pathog. 2025 Feb 7;21(2):e1012562. doi: 10.1371/journal.ppat.1012562 (PMC11805424; doi:10.1371/journal.ppat.1012562)

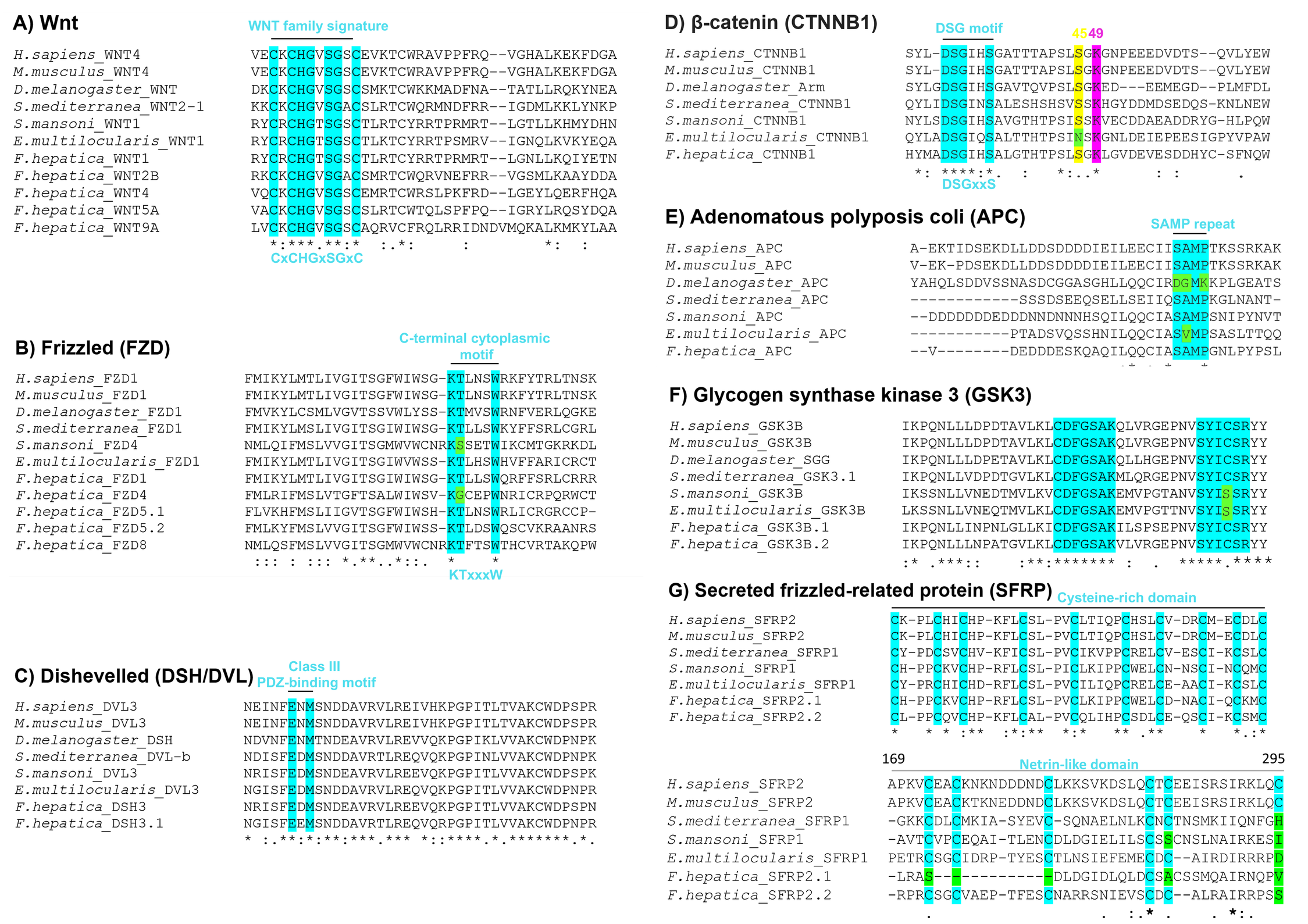

Supplement: S1 Fig — Multiple sequence alignments demonstrate the conservation of key functional domains in putative F. hepatica Wnt/β-catenin pathway components. A) Wnt family signature motif. B) C-terminal cytoplasmic motif of frizzled (FZD) receptors. C) Dishevelled class III PDZ-binding motif. D) β-catenin N-terminal residues essential for protein level regulation (light blue) and the conserved Ser45 (yellow) and Lys49 (magenta). DSG motif position denoted by black line. E) One of two adenomatous polyposis coli (APC) SAMP repeats. F) Two highly conserved motifs of the GSK-3 subfamily of serine/threonine protein kinases. G) SFRP cysteine rich domain and netrin-like domain. Residue positions relative to one another are not to scale. Sequence alignments were generated using Clustal Omega with default parameters. An asterisk (*) indicates a fully conserved amino acid, a colon (:) indicates conservation between strongly similar amino acids, a period (.) indicates conservation between weakly similar amino acids and a dash (-) indicates no consensus. Green denotes amino acid differences between the motifs of the species included. (TIF) [file ppat.1012562.s001.tif]

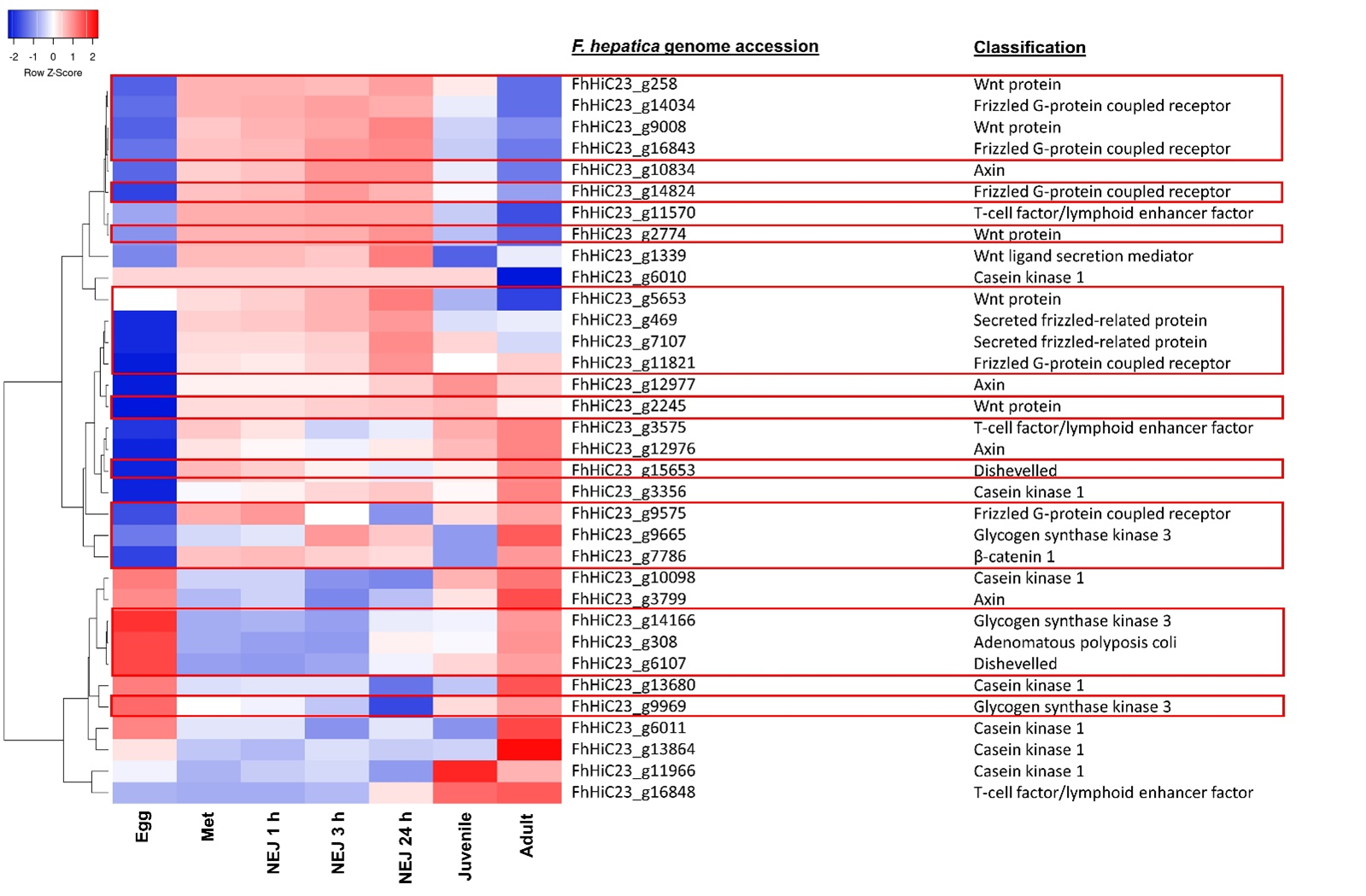

Supplement: S2 Fig — Developmentally staged expression heatmap generated from log2 TPM values of putative Wnt/β-catenin signalling pathway components. Columns correspond to life stages (egg; met – metacercariae; NEJ 1 h - newly-excysted juvenile 1 h post excystment; NEJ 3 h - NEJ 3 h post-excystment; NEJ 24 h - NEJ 24 h post-excystment; Juvenile – 3-week-old worms collected from murine livers; Adult - adult worms collected from the bile ducts of bovine livers). Each row corresponds to a different pathway component, as denoted by gene ID and annotation. Red boxes denote genes targeted in RNAi experiments. (TIF) [file ppat.1012562.s002.tif]

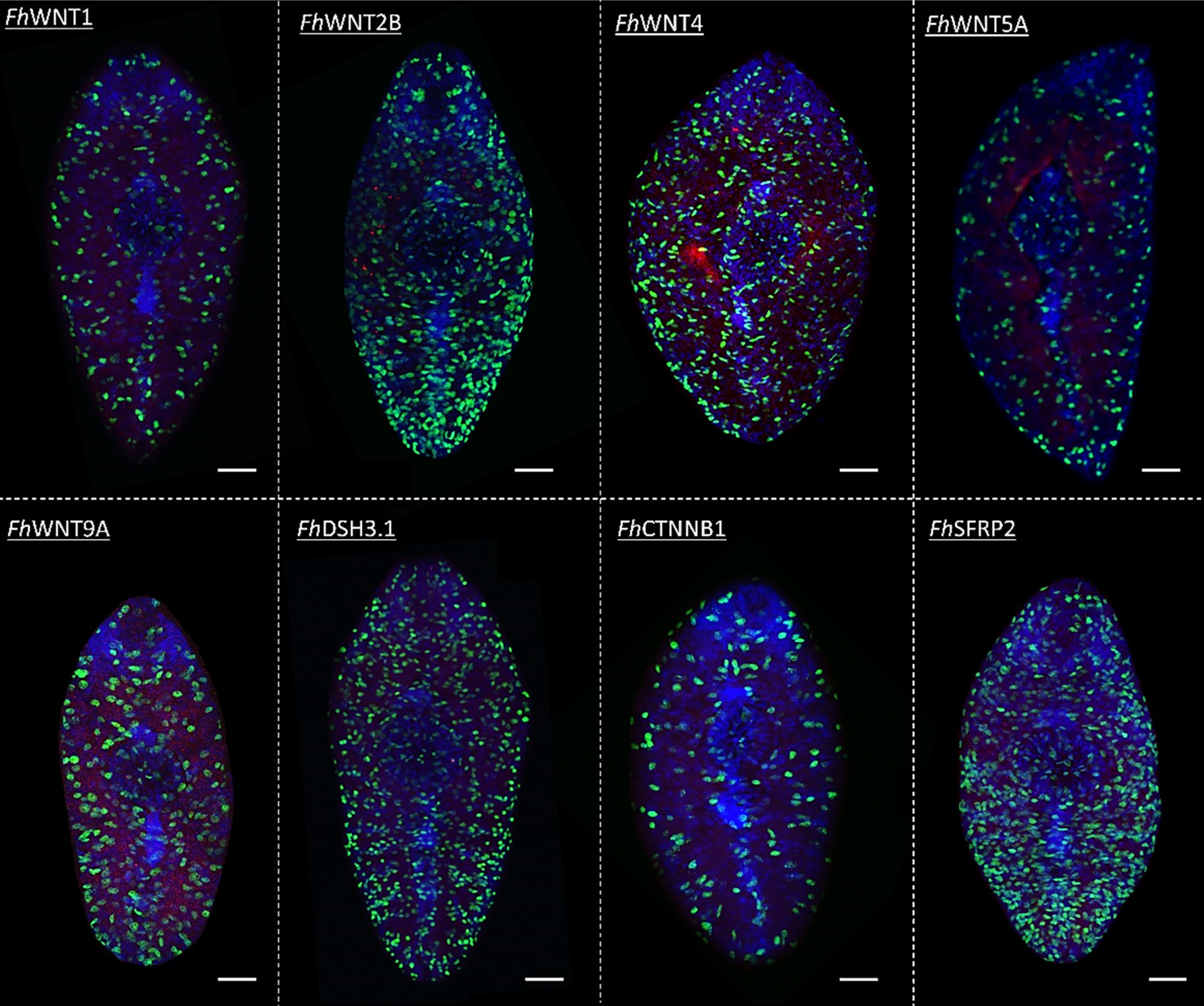

Supplement: S3 Fig — Fluorescence in situ hybridisation (FISH) negative control juvenile Fasciola hepatica exposed to sense (forward) strand RNA probes. Red (TAMRA) indicates non-specific binding, green fluorescence denotes EdU+ (neoblast-like) cells. DAPI (blue) served as a counterstain. Scale = 50 µm. (TIF) [file ppat.1012562.s003.tif]

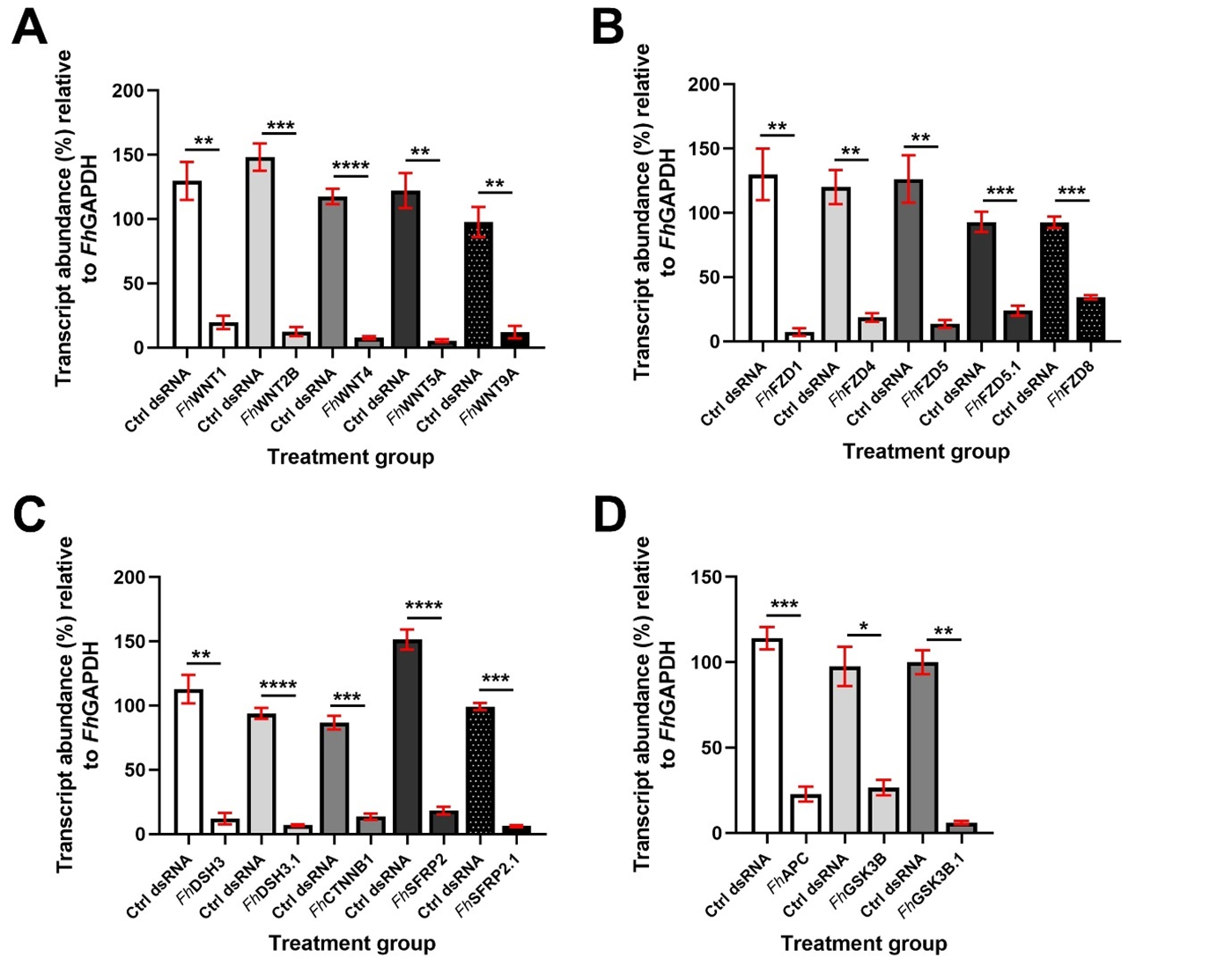

Supplement: S4 Fig — Transcript knockdown following RNA interference of Wnt/β-catenin pathway components in 4-week-old juvenile F. hepatica (A) FhWnts (B) FhFZDs (C) Other active Wnt signalling targets; FhDSH, FhCTNNB1 and FhSFRP (D) Destruction complex targets (n = 3). Data show mean expression (±SEM) of target transcript in control and target dsRNA-treated juveniles relative to untreated controls, using FhGAPDH as a housekeeping gene. Transcript knockdown was measured following nine 24-hour dsRNA exposures over a period of four weeks. Statistical analyses were performed using unpaired t-tests. *, p < 0.05; **, p < 0.01; ***, p < 0.001; ****, p < 0.0001. (TIF) [file ppat.1012562.s004.tif]

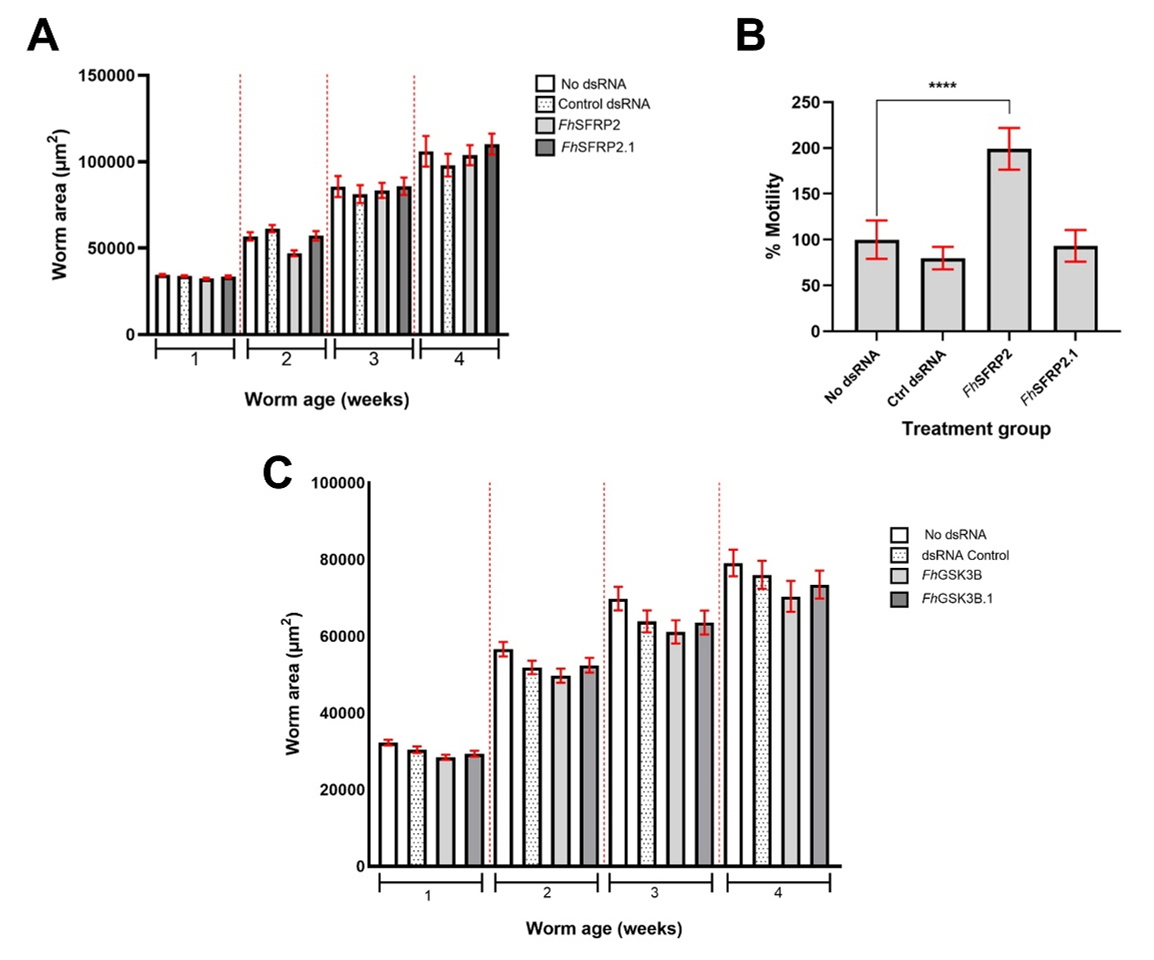

Supplement: S5 Fig — A) Growth of juvenile F. hepatica following RNAi-mediated silencing of FhSFRPs. B) Motility analysis of four-week old FhSFRP-silenced juveniles. C) Growth of juvenile F. hepatica following RNAi-mediated silencing of FhGSK3B. Worm area measured in μm2, data presented as μm2±SEM. Statistical analyses were performed using Kruskal Wallis with Dunn’s post hoc tests. ****, p < 0.0001. (TIF) [file ppat.1012562.s005.tif]

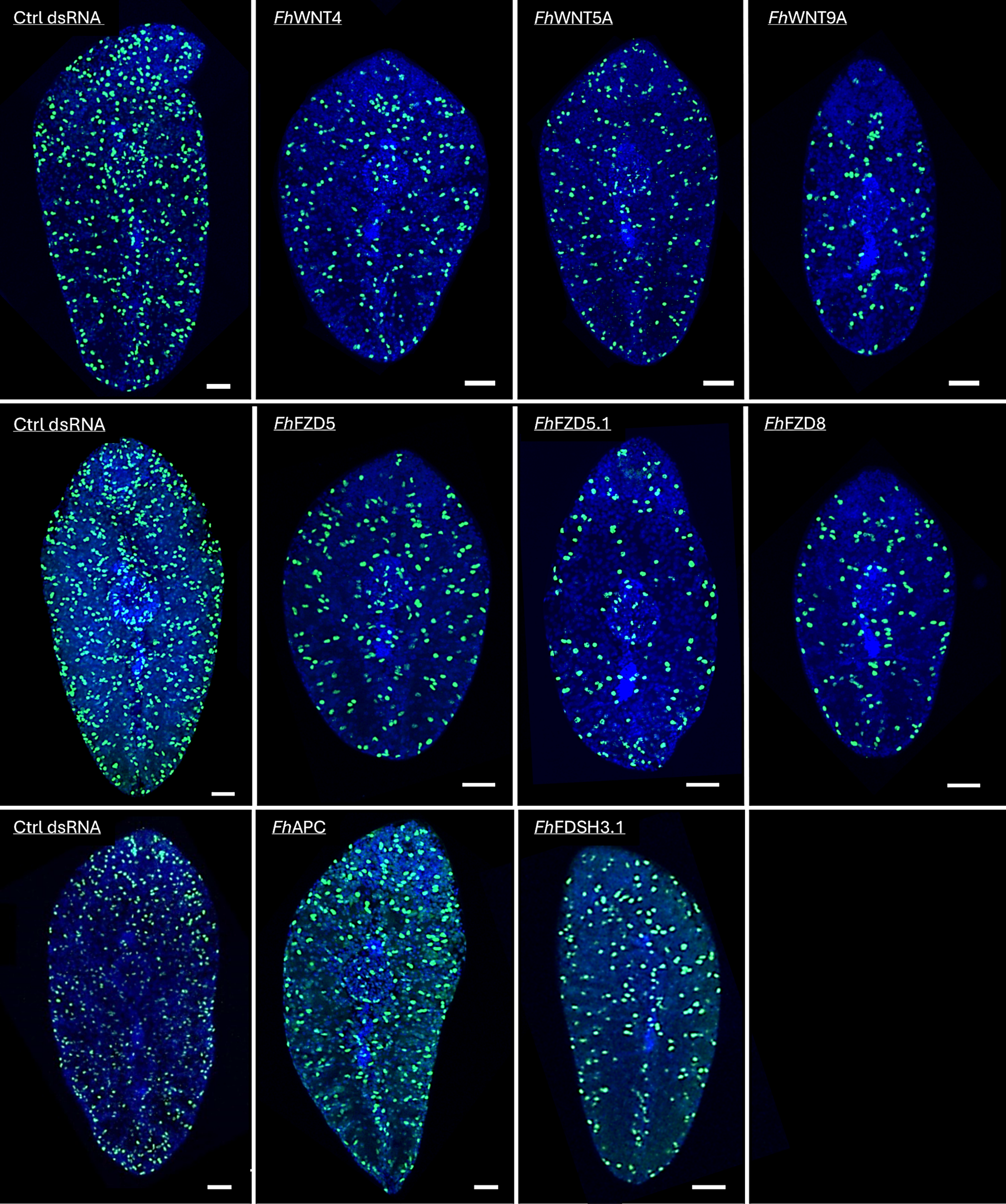

Supplement: S6 Fig — Maximally projected confocal z-stack images of EdU+ nuclei (green) in juvenile F. hepatica following four weeks of gene silencing. DAPI (blue) served as a counterstain. Scale = 50 µm. (TIF) [file ppat.1012562.s006.tif]

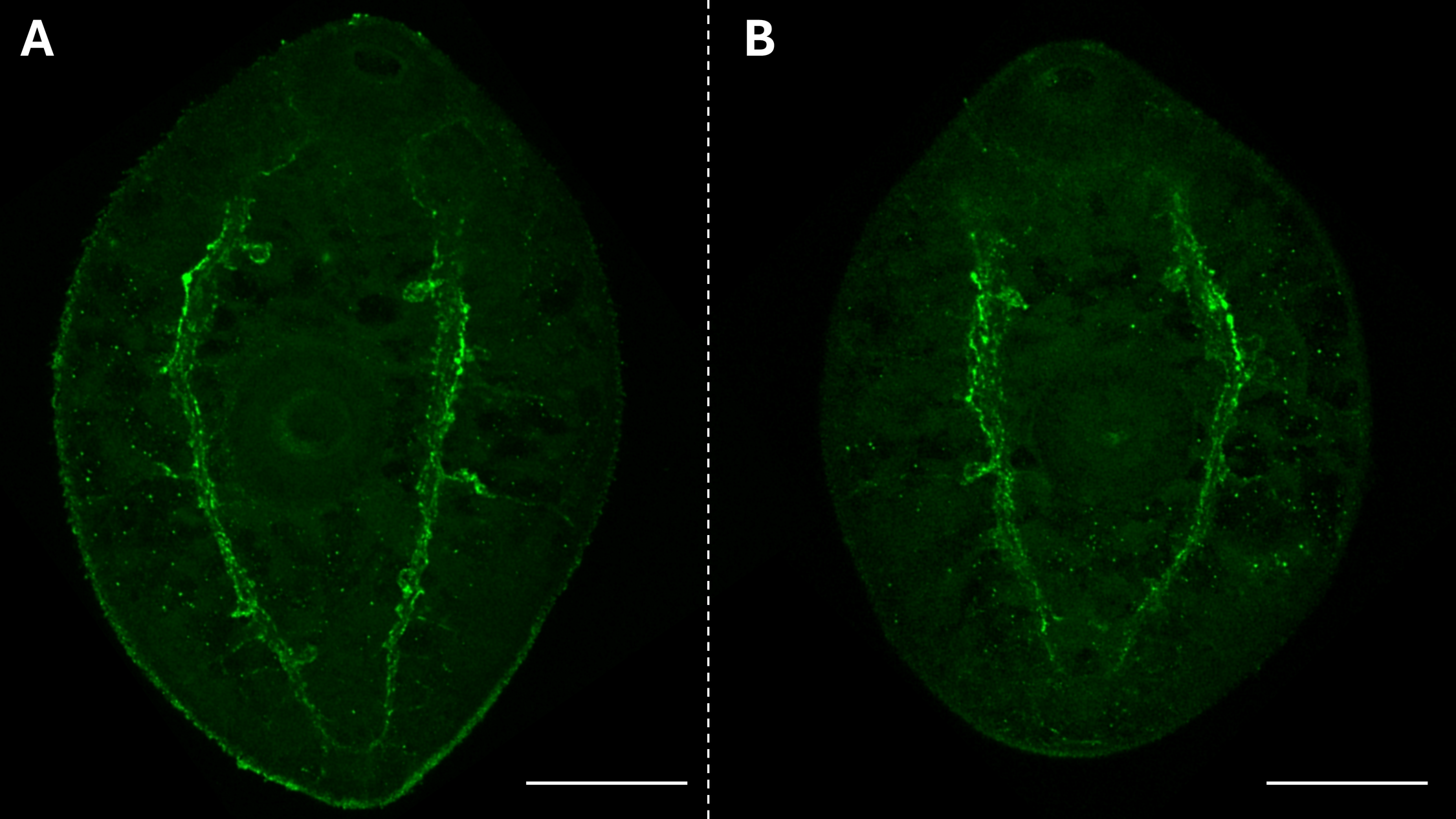

Supplement: S7 Fig — Confocal scanning laser micrographs of wholemount A) DMSO control and B) PP-treated two-week old F. hepatica subjected to ICC. Green staining denotes NPF immunoreactivity, highlighting the nervous system. Scale = 50 µm. (TIF) [file ppat.1012562.s007.tif]
