# Supplementary material for: Wnt/β-catenin signalling underpins juvenile Fasciola hepatica growth and development
Source: PLoS Pathog. 2025 Feb 7;21(2):e1012562. doi: 10.1371/journal.ppat.1012562 (PMC11805424; doi:10.1371/journal.ppat.1012562)
Supplement: S1 Table — Those pathway components lacking homologues in F. hepatica are denoted by an ‘X’. * Only present in PRJNA179522 genome assembly. (PDF) [file ppat.1012562.s008.pdf]

Pathway component	*F. hepatica* genome accession
**All pathways**	
**Wnt protein**	FhHiC23_g2774
	FhHiC23_g258
	FhHiC23_g9008
	FhHiC23_g5653
	FhHiC23_g2245
**Wnt Ligand Secretion Mediator (WLS)**	FhHiC23_g1339
**Frizzled GPCR**	FhHiC23_g9575
	FhHiC23_g11821
	FhHiC23_g14034
	FhHiC23_g16843
	FhHiC23_g14824
**Dishevelled (Dsh)**	FhHiC23_g6107
	FhHiC23_g15653
**Porcupine O-Acyltransferase (PORCN)***	D915_007339
**Wnt/β-catenin (canonical)**	
**β-catenin (CTNNB1)**	FhHiC23_g7786
**T-cell factor/lymphoid enhancer factor (TCF/LEF)**	FhHiC23_g16848
	FhHiC23_g11570
	FhHiC23_g3575
**Adenomatous polyposis coli (APC)**	FhHiC23_g14822
**Axin**	FhHiC23_g3799
	FhHiC23_g12977
	FhHiC23_g10834
	FhHiC23_g12976
	FhHiC23_g9665

**Glycogen synthase kinase 3β (GSK3β)**	FhHiC23_g9969
**Casein kinase 1 (CK1)**	FhHiC23_g11966
	FhHiC23_g3356
	FhHiC23_g13680
	FhHiC23_g13864
	FhHiC23_g10098
	FhHiC23_g6010
	FhHiC23_g6011
**B-cell CLL/lymphoma 9 protein (BCL9)**	X
**Pygopus**	X
**Wnt/calcium-dependent (non-canonical)**	
**Phospholipase C (PLC)**	FhHiC23_g1854
	FhHiC23_g8107
**Ca2+/calmodulin-dependent protein kinase II (CaMKII)**	FhHiC23_g12764
	FhHiC23_g3316
	FhHiC23_g13019
**Protein kinase C (PKC)**	FhHiC23_g182
	FhHiC23_g15159
	FhHiC23_g12334
	FhHiC23_g12169
**Calcineurin**	FhHiC23_g16682
**Nuclear factor of activated T-cells (NFAT)**	X
**Cell division control protein 42 homolog (CDC42)**	X
**Wnt/planar cell polarity (non-canonical)**	
**Rho GTPase**	FhHiC23_g44
	FhHiC23_g6148
	FhHiC23_g9840
**c-Jun N-terminal kinase (JNK)**	FhHiC23_g16129

**Rho-associated, coiled-coil-containing protein kinase (ROCK)**	FhHiC23_g1890
**Disheveled-associated activator of morphogenesis 1 (DAAM1)**	X
**Antagonists**	
**Secreted frizzled-related protein (SFRP)**	FhHiC23_g7107
	FhHiC23_g469
**Wnt inhibitory factor (WIF)**	X
**Dikkopf**	X
**Cerberus**	X
