# Supplementary material for: Wnt/β-catenin signalling underpins juvenile Fasciola hepatica growth and development
Source: PLoS Pathog. 2025 Feb 7;21(2):e1012562. doi: 10.1371/journal.ppat.1012562 (PMC11805424; doi:10.1371/journal.ppat.1012562)
Supplement: S2 Table — N.B. qPCR reverse primers were used in conjunction with the corresponding dsRNA forward primer. (PDF) [file ppat.1012562.s009.pdf]

S2 Table. Oligonucleotide primers used in double stranded (ds)RNA synthesis, qPCR analyses and fluorescence *in situ* hybridisation (FISH) of *Fh*Wnt pathway targets. N.B. qPCR reverse primers were used in conjunction with the corresponding dsRNA forward primer.

**Gene of interest**	**Primer sequence Forward/Reverse (F/R - 5'-3')**	**Amplicon size (bp)**
*Fh*FZD1<br>ID: FhHiC23_g9575	dsRNA Synthesis	
	F: taatacgactcactatagggtATTTACATTTTGCCGCTTGG	177
	R: taatacgactcactatagggtATCCCGTGCCAATCACTAAG	
	qPCR	
	R: TTGAGCCTCCAGTTTTGATG	240
	FISH	
	F: taatacgactcactatagggtGGCCGATTATCTTCCTCTCC	1012
	R: taatacgactcactatagggtCTGTCAACCGGAAGACCAAT	
*Fh*FZD4<br>ID: FhHiC23_g16843	dsRNA Synthesis	
	F: taatacgactcactatagggtCACGGGAATGGATTTACCAC	197
	R: taatacgactcactatagggtAAACAGAACAGCCCCATACG	
	qPCR	
	R: GGACTGGGACCGACAACTT	200
	FISH	
	F: taatacgactcactatagggt TGCTAGTTCGATCTGGTGGA	1000
	R: taatacgactcactatagggt ACGTAACCAACCGTTTGAGG	
*Fh*FZD5<br>ID: FhHiC23_g14034	dsRNA Synthesis	
	F: taatacgactcactatagggtAAGCACAGAACGCCATATCC	199
	R: taatacgactcactatagggtACATTGAACGCCGGTAGAAC	
	qPCR	
	R: ATTCCGTGGATCGAACTGTC	249
	FISH	
	F: taatacgactcactatagggtCGTGTAGTGGACGAGTCAGC	1000
	R: taatacgactcactatagggtGACGTTCCGGGTACTGAAAA	
*Fh*FZD5.1<br>ID: FhHiC23_g14824	dsRNA Synthesis	
	F: taatacgactcactatagggtGTGGCCACTATGACCGACTT	174

	R: taatacgactcactatagggtTACACGACGGTCACCGATTA	
	qPCR	
	R: ACAGCAGAAACGGCTGACTC	200
	FISH	
	F: taatacgactcactatagggtTTGGTTTATGGGCTGTCCTG	1000
	R: taatacgactcactatagggtCGATTAGCCGCTCGTTTTAC	
*Fh*FZD8<br>ID: FhHiC23_g11821	dsRNA Synthesis	
	F: taatacgactcactatagggtTTCTCAAGGACCCGTACCAC	194
	R: taatacgactcactatagggtGTTCATGAATGGGCACACAC	
	qPCR	
	R: TGCATATTGTATGCCTTGACAG	220
	FISH	
	F: taatacgactcactatagggtTTCTCAAGGACCCGTACCAC	1000
	R: taatacgactcactatagggtGGCACGATCCAAAATGAATG	
*Fh*WNT1<br>ID: FhHiC23_g9008	dsRNA Synthesis	
	F: taatacgactcactatagggtATAAGGTTGGACGGCAGTTG	163
	R: taatacgactcactatagggtATGCGGATTGAATTGGTCTC	
	qPCR	
	R: TGCTTTGCGGAATTTTGTATC	230
	FISH	
	F: taatacgactcactatagggtGACAATGCCACACAAACCAG	832
	R: taatacgactcactatagggtACTGCCGTCCAACCTTATTG	
*Fh*WNT2B<br>ID: FhHiC23_g2774	dsRNA Synthesis	
	F: taatacgactcactatagggtTCCCGGATCGGAGTAGTATG	229
	R: taatacgactcactatagggtACTGATGCGCTGTTTGTCTG	
	qPCR	
	R: TCCGCTAACTTTCGGAACTG	245
	FISH	

	F: taatacgactcactatagggt CAGACAAACAGCGCATCAGT	1000
	R: taatacgactcactatagggt ATCCACTACCGACTCCACCA	
*Fh*WNT4<br>ID: FhHiC23_g5653	dsRNA Synthesis	
	F: taatacgactcactatagggtCCTGCACAATAACCATGTCG	168
	R: taatacgactcactatagggtAAAATGGCTTGATGGAAACG	
	qPCR	
	R: AATTGGTGATCCACCGTGTC	222
	FISH	
	F: taatacgactcactatagggtTCTTGCGAAATGAGAACGTG	1000
	R:taatacgactcactatagggtCAGTGTAGTCATCCTGCGACA	
*Fh*WNT5A<br>ID: FhHiC23_g258	dsRNA Synthesis	
	F: taatacgactcactatagggtTTGGCCTATCTGGACGAATC	205
	R: taatacgactcactatagggtACTCAACACGACAGGACCAG	
	qPCR	
	R: CGCACACAGGTCTGACACTC	221
	FISH	
	F: taatacgactcactatagggt TCTGACAGGCAGTCGTGAAG	1000
	R: taatacgactcactatagggt TGATGATTGGAACTGGATGC	
*Fh*WNT9A<br>ID: FhHiC23_g2245	dsRNA Synthesis	
	F: taatacgactcactatagggtGGATAAAAACAGCGCTTTGG	179
	R: taatacgactcactatagggtACCCGAGAAAAGCTGATGTG	
	qPCR	
	R: GCTGTACCGACAGAACACGA	209
	FISH	
	F: taatacgactcactatagggtAGAACGCTGCAATTCGAAAG	1000
	R: taatacgactcactatagggtGCTGTACCGACAGAACACGA	
*Fh*DSH3<br>ID: FhHiC23_g6107	dsRNA Synthesis	
	F: taatacgactcactatagggtCTACGGGGACGTCAATGTCT	192
	R: taatacgactcactatagggtTGATGCCGAGGAAGTTTACC	

	qPCR	
	R: CCCACATAGATCCCACCATC	241
*Fh*DSH3.1<br>ID: FhHiC23_g15653	dsRNA Synthesis	
	F: taatacgactcactatagggtAACAAACGGGTCTGGATCTG	226
	R: taatacgactcactatagggtTGAAGCGTGATCGTGTTAGC	
	qPCR	
	Reverse: GTGTCGCGAGTCAAAAATGA	272
	FISH	
	F: taatacgactcactatagggtAATGGGGTCTCCGGTTATTC	861
	R: taatacgactcactatagggtTGAAGCGTGATCGTGTTAGC	
*Fh*CTNNB1<br>ID: FhHiC23_g7786	dsRNA Synthesis	
	F: taatacgactcactatagggtCTGGGGGTCGATGAAGTAGA	245
	R: taatacgactcactatagggtCTAGCGGCCTCGTCTGTATC	
	qPCR	
	R: TCTTCATCTTTTATTAGCTTCACCA	284
	FISH	
	F: taatacgactcactatagggtGACCAATTGTGTTCCCTGCT	838
	R: taatacgactcactatagggtAAGACCGAGCTCCTGACAAA	
*Fh*APC<br>ID: FhHiC23_g308	dsRNA Synthesis	
	F: taatacgactcactatagggtCTAACACCCGCGTCCATAGT	200
	R: taatacgactcactatagggtTCTGAATGGGGCGATTTTAC	
	qPCR	
	R: AGCACGATGCACAACAACTC	267
*Fh*GSK3B<br>ID: FhHiC23_g9969	dsRNA Synthesis	
	F: taatacgactcactatagggtTAGCTCCCGTTCCTTCAAGA	196
	R: taatacgactcactatagggtACTCGTTCACAGTGGGGAAG	
	qPCR	
	R: CGCACTGGTGGTTAGACAGA	267
*Fh*GSK3B.1<br>ID: FhHiC23_g14166	dsRNA Synthesis	
	F: taatacgactcactatagggtTTGATATTTGGTGCGGTTGA	212
	R: taatacgactcactatagggtCGAATTTGAGGGAATCGAAA	

	qPCR	
	R: CGGACGGAAAACCTTAGACC	243
*Fh*SFRP2<br>ID: FhHiC23_g469	dsRNA Synthesis	
	F: taatacgactcactatagggtGCTCCAGTCTCGAGGAAATG	247
	R: taatacgactcactatagggtACTTGGGATTGGTCAACAGC	
	qPCR	
	R: AACCCATACTTGGGATTGGTC	254
	FISH	
	F: taatacgactcactatagggtGGCTCCAGTCTCGAGGAAAT	1000
	R: taatacgactcactatagggtGAGGTGGATCAATGCTGACA	
*Fh*SFRP2.1<br>ID: FhHiC23_g7107	dsRNA Synthesis	
	F: taatacgactcactatagggtGAATAACCGAGCTGCTTTGC	224
	R: taatacgactcactatagggtTATCATTGCTGCCGTAGTCG	
	qPCR	
	R: GTTTGCCCCTTTATCATTGC	235
Neomycin phosphotransferase	dsRNA Synthesis	
	F: taatacgactcactatagggGGTGGAGAGGCTATTCGGCT	200
	R: taatacgactcactatagggCCTTCCCGCTTCAGTGACAA	
*Fh*GAPDH<br>ID: FhHiC23_g5828	qPCR	
	F: GATTTTAAAGTCGCGCTTCG	
	R: ACATCAACGATGCCCTTCTC	215
*Fh*MLC<br>ID: FhHiC23_g308	qPCR	
	F: AACGGGTTACCTTCGAGACC	193
	R: TGGTCCACTTGATGTTCGTC	
*Fh*Actin<br>ID: FhHiC23_g16496	qPCR	
	F: GGGTATGTGCAAAGCTGGAT	200
	R: CATCCCAGTTCGTGACAATG	
