# Supplementary material for: Wnt/β-catenin signalling underpins juvenile Fasciola hepatica growth and development
Source: PLoS Pathog. 2025 Feb 7;21(2):e1012562. doi: 10.1371/journal.ppat.1012562 (PMC11805424; doi:10.1371/journal.ppat.1012562)
Supplement: S3 Table — (PDF) [file ppat.1012562.s010.pdf]

***F. hepatica* genome accession**	**Top hit: *H. sapiens* (taxid: 9606)**	**Top hit: *M. musculus* (taxid: 10090)**	**Top hit: *S. mediterranea* (taxid: 79327)**	**Top hit: *D. melanogaster* (taxid: 7227)**	**Top hit: *S. mansoni* (taxid: 6183)**	**Top hit: *E. multilocularis* (taxid: 6211)**
FhHiC23_g9008	Proto-oncogene Wnt-1	Protein Wnt-10A	Protein Wnt-1	Wingless	Smp_248080	EmuJ_000349900
FhHiC23_g2774	Protein Wnt-2b	Protein Wnt-10B	Protein Wnt-2-1	Wingless	Smp_167140	EmuJ_000748600
FhHiC23_g5653	Protein Wnt-4	Protein Wnt-4	Protein Wnt-a	Wnt-2 protein	Smp_332550	EmuJ_000211300
FhHiC23_g258	Protein Wnt-5a	Protein Wnt-5B	Protein Wnt-5	Wnt-2 protein	Smp_145140	EmuJ_000804000
FhHiC23_g2245	Protein Wnt-9a	Protein Wnt-9a	Protein Wnt-11-1	Protein Wnt-5	Smp_156540	EmuJ_000907500
FhHiC23_g9575	Frizzled-1	Frizzled-1	Frizzled-1	Frizzled class receptor	frizzled class receptor 1	EmuJ_000682100
FhHiC23_g16843	Frizzled-4	Frizzled-4	Frizzled-4	Frizzled-4	Smp_247930	EmuJ_000085700
FhHiC23_g14034	Frizzled-5	Frizzled-8	Frizzled 5/8-3	FBgn0001085	Smp_155340	EmuJ_000996400
FhHiC23_g14824	Frizzled-5	Frizzled-8	Frizzled 5/8-4	Frizzled-2	Smp_139180	EmuJ_000996400
FhHiC23_g11821	Frizzled-8	Frizzled-8	Frizzled-4	Frizzled-2	Smp_174350	EmuJ_000438200
FhHiC23_g6107	Dishevelled segment polarity protein 3	Dishevelled segment polarity protein 3	Dishevelled like protein, DVL-b	Dishevelled segment polarity protein	Smp_020300	EmuJ_000118000
FhHiC23_g15653	Dishevelled segment polarity protein 3	Dishevelled segment polarity protein 3	Dishevelled like protein, DVL-b	Dishevelled segment polarity protein	Smp_347960	EmuJ_000423700
FhHiC23_g7786	Catenin Beta 1	Catenin Beta 1	Catenin Beta 1	Armadillo	Smp_023550	EmuJ_001007700
FhHiC23_g9969	Glycogen synthase kinase 3-beta	Glycogen synthase kinase 3-beta	Glycogen synthase kinase 3.1	Gasket	Smp_008260	EmuJ_000352700
FhHiC23_g14166	Glycogen synthase kinase 3-beta	Glycogen synthase kinase 3-beta	Glycogen synthase kinase 3.1	Shaggy	Smp_008260	EmuJ_000980300
FhHiC23_g7107	Secreted Frizzled-related protein 2	Secreted Frizzled-related protein 2	Secreted Frizzled-related protein 1	Frizzled gene exon 1	Smp_068680	EmuJ_000838700
FhHiC23_g469	Secreted Frizzled-related protein 2	Secreted Frizzled-related protein 2	Secreted Frizzled-related protein 1	Frizzled gene exon 1	Smp_062560	EmuJ_000838700
FhHiC23_g14822	Adenomatous polyposis coli	Chain A, Adenomatous polyposis coli protein	Adenomatous polyposis coli-like protein	Adenomatous polyposis coli	Smp_343270	EmuJ_001070700
